# Diversity and functional analysis of light‐driven pumping rhodopsins in marine Flavobacteria

**DOI:** 10.1002/mbo3.321

**Published:** 2015-12-13

**Authors:** Yong Min Kwon, So‐Young Kim, Kwang‐Hwan Jung, Sang‐Jin Kim

**Affiliations:** ^1^ Marine Biotechnology Research CenterKorea Institute of Ocean Science & Technology787 HaeanroAnsan426‐744Korea; ^2^ Department of Life Science and Institute of Biological ScienceSogang University35 Baekbeom‐RoMapo‐GuSeoul121‐742Korea; ^3^ Marine Biodiversity Institute of KoreaSeocheon325‐902Korea

**Keywords:** Chloride pumping rhodopsin, light‐driven pump activity, marine flavobacteria, proteorhodopsin, sodium pumping rhodopsin

## Abstract

The aims of this study are the description of diversity for proteorhodopsin (PR)‐containing flavobacteria in marine environments, the finding of novel photoreceptive membrane proteins, and the elucidation of the effect of light on the growth of three rhodopsin genes containing flavobacterium. We investigated novel sodium ion rhodopsin (NaR) and halorhodopsin (HR) genes from PR‐containing flavobacteria that were previously isolated from diverse aquatic sites, mainly from tidal flat sediment (62.5%). In 16 PR‐containing isolates, three new types of genes were found. Among these three isolates, one (*Nonlabens* sp. YIK11 isolated from sediment) contained both the NaR and chloride ion rhodopsin (ClR) ‐ HR type of gene. The sequences showed that the DTE (proton pump), NDQ (sodium ion pump) and NTQ (chloride ion pump) motifs corresponding to the D85, T89, and D96 positions in bacteriorhodopsin (BR) were well conserved. Phylogenetic analysis indicated that three NaR and one ClR grouped within the same clade, as previously reported. Illumination of cell suspensions showed the change in proton pump activity, supporting that one or more rhodopsins are functional. The qRT‐PCR study revealed that three rhodopsin genes, especially NaR, are highly induced when they are incubated in the presence of light or in the absence of sufficient nutrients. The expression levels of the DTE, NDQ, and NTQ motif‐containing rhodopsin genes in YIK11 correlate positively with illumination, but negatively with nutrient levels. Based on those results, we concluded that light has a positive impact on the relative expression levels of the three rhodopsin genes in the flavobacterium, *Nonlabens* sp. YIK11, but with no apparent positive impact on growth. Consequently, light did not stimulate the growth of YIK11 as determined by cell numbers in a nutrient‐limited or ‐enriched medium, although it contains and induces three rhodopsins.

## Introduction

Rhodopsins are a family of membrane‐embedded photoactive retinylidene proteins discovered in the three domains of life and typically have seven transmembrane *α*‐helix domains, which covalently bind to light‐absorbing all‐trans retinal (Spudich et al. 2000). Microbial rhodopsins classified as type I were first found in the extremely halophilic archaea. Bacteriorhodopsin (BR) and halorhodopsin (HR) function as a light‐driven outward proton pump and inward chloride pump, respectively, (Oesterhelt and Stoeckenius 1971; Schobert and Lanyi 1982) and the other two halobacterial sensory rhodopsin I and II (SRI and SRII) function as light‐activated signal transducers (Bogomolni and Spudich 1982; Spudich and Spudich 1982). In the bacteria, proteorhodopsin (PR) functioning as a light‐driven proton pump was first discovered from an uncultured *γ‐proteobacterium* of the SAR86 group, and it was suggested that PR plays an important role in the marine ecosystem by providing energy for microbial metabolism (Béjà et al. 2000, 2001). Since then, PR genes have been found in various aquatic environments, including seawater, freshwater, and brackish water (de la Torre et al. 2003; Sabehi et al. 2004; Venter et al. 2004; Giovannoni et al. 2005; Frigaard et al. 2006; Atamna‐Ismaeel et al. 2008; Campbell et al. 2008; Koh et al. 2010; Yoshizawa et al. 2012), and they are one of the most highly expressed and widely distributed proteins in marine bacterial communities (Frias‐Lopez et al. 2008). On the basis of genomic survey data, PRs appear in 13–80% of marine bacteria and archaea in oceanic surface seawaters (de la Torre et al. 2003; Moran and Miller 2007). The recent discovery of a new type of microbial rhodopsin (sodium ion pumping rhodopsin; NaR) came after analysis of whole‐genome sequences of the PR‐containing marine flavobacteria, *Nonlabens dokdonensis* DSW‐6^T^, *Krokinobacter eikastus* NBRC 100814, *Nonlabens marinus* S1‐08^T^, and *Gillisia limnaea* R‐8282^T^ (Inoue et al. 2013; Kwon et al. 2013; Balashov et al. 2014; Yoshizawa et al. 2014). Furthermore, another new type of microbial rhodopsin (chloride ion pumping rhodopsin; ClR), similar to HR, was recently discovered from the PR‐ and NaR‐containing *N*.* marinus* S1‐08^T^ (Yoshizawa et al. 2014). Interestingly, a marine flavobacterium, *N. marinus* S1‐08^T^, encoding three different types of microbial rhodopsins is unique in having multiple functions of light‐driven inward or outward translocating, H^+^, Na^+^, and Cl^−^ pumps in one cell. The authors demonstrated that growth of S1‐08^T^ is markedly stimulated by light under carbon‐limited conditions, such as in *Dokdonia* sp. MED134 (Gómez‐Consarnau et al. 2007). In addition, light‐enhanced growth was reported in *Psychroflexus torques* ATCC 700755^T^ under salinity stress conditions (Feng et al. 2013), and starved *Vibrio* sp. AND4 had a more rapid growth recovery under light (Gómez‐Consarnau et al. 2010). In contrast, light‐enhanced growth in PR‐containing bacteria did not produce consistent results (Giovannoni et al. 2005; Stingl et al. 2007; González et al. 2008; Steindler et al. 2011; Riedel et al. 2013).

In this study, we report the presence and function of one or more microbial rhodopsin‐containing isolates from diverse marine environments such as sediment (previously unreported), algae, a marine animal, and a glacier. The rhodopsin‐mediated pump activity of each isolate was directly observed from the native cells. The expression of each of the genes and growth experiments under diverse culture conditions were investigated to determine the effect of light on *Nonlabens* sp. YIK11, encoding three different types of rhodopsin in the one cell.

## Experimental Procedures

### Bacterial isolation and culture conditions

Bacteria were isolated from tidal flat sediments (Kwon et al. 2014b), unidentified marine sponges, unknown algae, and a glacier ice core (Kwon et al. 2014a). Strains collected from each sample were isolated according to the procedure described by Bae et al. (2007), Kwon et al. (2014a,b) and Yang et al. (2006). Strains were grown under aerobic conditions and produced orange‐ or yellow‐colored carotenoid pigments. The isolates were routinely cultured on Marine Broth (MB; Difco, Detroit, USA) with 1.5% agar or ZoBell e2216 medium (ZB; 5 g peptone, 1 g yeast extract, 0.01 g FePO_4_ per liter of 20% distilled water, and 80% aged seawater) at optimal temperatures (Table 1). Seawater for experiments was collected at the East Sea station (37°00.000′N, 131°00.000′E) from the surface layer. *Nonlabens marinus* (basonym: *N. marina*) S1‐08^T^ was provided by Dr. S. Park et al. (2012).

**Table 1:** Proteorhodopsin, sodium, and chloride ion rhodopsin‐containing isolates used in this study

Isolate	Sample	Optimal Temp (°C)	Location	Coordinates	Accession number
					16S
*Nonlabens* sp. YIK11^a^	Sediment	30	Youngheung Island, Korea	37°16′N 126°26′E	JX312332
*Hoppeia youngheungensis* YIK12^T^					JX312333
*Sediminicola* sp. YIK13					JX312334
*Sediminicola* sp. YIK23					JX312335
*Sediminicola* sp. TAK34	Marine animal	30	Taean, Korea	36°46′N 126°33′E	JX312336
*Flavobacterium* sp. TAK38	Sediment				JX312337
*Dokdonia sp*. TIK166	Algae	30	Taejongdae Island, Korea	34°53′N 128°57′E	JX312341
*Dokdonia* sp. DIK51	Sediment	30	Daebu Island, Korea	37°17′N 126°37′E	JX312338
*Aquimarina* sp. DIK55					JX312339
*Maribacter* sp. DIK293					JX312342
*Dokdonia sp*. HJK127	Sediment	25	Hujin, Korea	37°28′N 129°10′E	JX312340
*Flagellimonas sp*. DK169	Algae	25	Dokdo Island, Korea	37°14′N 131°52′E	KM461123
*Nonlabens sp*. MIC269^a^	Sediment	30	Chuuk Lagoon, Micronesia	07°25'N 44”151°51'32”E	KJ019870
*Nonlabens antacticus* AKS622^T^	Glacier	15	King George Island, Antarctica	62°13′S 58°47′W	DQ660393
*Sediminicola sp*. SOR394	Marine animal	30	Okhotsk, Russia	54°22′N 134°59′E	JX312343
*Nonlabens marinus* S1‐08^T^ a ^,^ b	Seawater	20	Western North Pacific Ocean	30°11′N 145°05′E	AB602426

a Strains possessing the two or three types of rhodopsins.

b S1‐08^T^ (Park et al. 2012).

### Identification of the PR, NaR, and ClR genes

Genomic DNA was extracted by using an Exgene DNA extraction kit (Gene All, Seoul, Korea) and the PR gene was amplified under the PCR conditions described by Yoshizawa et al. (2012). The NaR (amino acid positions 93–307 and 212–307 based on DSW‐6^T^ numbering) and ClR (amino acid positions 57–208 based on S1‐08^T^ numbering) genes were amplified using designed degenerate primers based on the conserved amino acid sequences of flavobacteria or other taxa found in the National Center for Biotechnology Information (NCBI). The primers used in this study are shown in Table S1. DNA‐free Taq (CellSafe, Suwon, Korea) was used for PCR amplification under the following conditions: one cycle of 95°C for 5 min; 40 cycles of 95°C for 30 sec, 55°C for 30 sec, and 72°C for 1 min; and finally one cycle of 72°C for 10 min. The amplified PCR products were separated on a 1% agarose gel and these fragments were purified, cloned into the pGEM T‐easy vector (Promega, Madison, WI, USA), and sequenced. The complete base sequences of the PR, NaR, and ClR genes from this sequenced fragment were obtained by PCR using the DNA Walking SpeedUp Premix kit (Seegene, Seoul, Korea) according to the manufacturer's specifications. This kit was used to obtain unknown sequences that lie adjacent to the known sequence of the PCR product obtained above. This PCR technique was employed to directly amplify unknown sequences using six target‐specific primers in the known sequence and DNA walking‐annealing control primers and a universal primer in the unknown sequence region, which was designed by the manufacturer to capture unknown target sites with high specificity. These fragments were purified and cloned into the pGEM T‐easy vector and then sequenced. The 16s rRNA gene sequences obtained in this study were deposited in the NCBI database and the accession numbers are listed in Table 1.

### Analysis of sequence data

The complete coding sequences of PR, NaR, and ClR were compared with the GenBank database using the program BLASTX. The related amino acid sequences retrieved from NCBI were used for sequence alignment and phylogenetic analysis. Editing and analysis of the sequence data were performed using Clustal W of DNASTAT (DNASTAR Inc., Madison, WI, USA) and MEGA 5 software (Tamura et al. 2011). Phylogenetic trees were constructed by the neighbor‐joining method based on the Jukes & Cantor and JTT matrix distance model, with 1000‐replicate bootstrap analyses for statistical support (Felsenstein 1985).

### Measurement of light‐driven pump activity of microbial isolates

Strains were grown in MB or ZB medium in a shaking incubator at 150 rpm and their growth was monitored by measuring the optical density of cultures at 600 nm (OD_600_). Cells were harvested by centrifugation at 6000× ***g*** for 15 min at 4°C, washed twice with artificial seawater (ASW) (Oh et al. 1991), and resuspended in ASW to a concentration of 3 × 10^9^ cells mL^−1^. Two milliliters of cell suspensions were first placed in darkness and then illuminated at 100 W m^−2^ through a short‐wave cutoff filter (>440 nm, Sigma Koki SCF‐50S‐44Y, Saitama, Japan) in combination with a focusing convex lens and heat‐protecting (1% CuSO_4_ solution) filter. The pH values were monitored with a computerized pH meter (Horiba F‐51, Japan). Measurements were repeated under the same conditions after addition of uncoupling agent carbonyl cyanide m‐chlorophenylhydrazone (CCCP) to a final concentration of 10 *μ*mol L^−1^ and further addition of tetraphenylphosphonium bromide (TPP^+^) to a final concentration of 30 mmol L^−1^.

### Growth experiments of YIK11

Experiments were carried out to determine the effect of light under varying salinity, and under different nutrient levels. The effect of nutrient conditions was examined in aged seawater containing dissolved organic carbon (DOC) in a series of concentrations (0.14, 0.39, and 0.74 mmol L^−1^ C) with 225 *μ*mol L^−1^ NH_4_Cl and 44.7 *μ*mol L^−1^ Na_2_HPO_4_·H_2_O according to the procedure described by Kimura et al. (2011) and Gómez‐Consarnau et al. (2007), and in ZB medium. DOC concentrations were measured with a TOC‐V total carbon analyzer (Shimadzu, Japan). The effect of salinity conditions was examined in ZB medium containing a series of different salt concentrations (1.5, optimum 3 and 8%) and grown in either green light or in the dark. During growth, the YIK11 cells in this experiment were constantly exposed to light from a green‐light‐emitting diode (LED, Aua Illumination) at intensities of ca. 160 and 25 *μ*mol photons m^−2^ s^−1^. DOC medium (500 mL) and ZB medium (100 mL) were inoculated with 1% working culture (1 × 10^6^ CFU mL^−1^) and incubated at 30°C. All culture experiments were performed in triplicate.

### Bacterial cell number

To estimate total bacterial numbers, we fixed samples in 2.5% (w/v) glutaraldehyde and analyzed them using a qNano instrument (Izon Science, Christchurch, New Zealand) according to the manufacturer's specifications. The instrument is based on the Coulter principle at the nanoscale, and operates by detecting transient changes in the ionic current generated by the transport of the target particles through a size‐tunable nanopore (NP) in a thick thermoplastic polyurethane membrane. Cells were diluted 1000‐fold into 0.2 *μ*m‐filtered 1X TBT (100 mmol L^−1^ Tris‐HCl, 100 mmol L^−1^ NaCl and 10 mmol L^−1^ MgCl_2_, pH 7.4) buffer and detected using an NP1000 (size range of 500 to 2000 nm) membrane. Voltage was adjusted until the current reached approximately 130–150 nA and samples were loaded. Each measurement recorded at least 500 pulses, and these measurements were typically repeated three times. The standard calibration particles (CP1000, a stock concentration of 5.5 × 10^10^ mL^−1^) were measured under the same conditions as the calibration control. Data were digitized and interpreted using Izon control software (version 2.2).

### RNA extraction

Cells of YIK11 were cultured as described above and 3 mL of the culture were harvested via centrifugation at 13,000× ***g*** for 5 min within the exponential, early, and late stationary phases. The cell pellet was stored immediately in 500 *μ*L of RNAlater (Ambion, Austin, TX, USA) and kept at −20°C until RNA extraction. Total RNA was extracted using an RNease mini kit (Qiagen, Valencia, CA, USA) according to manufacturer's instructions. In addition, an on‐column DNase treatment using RNase‐free DNase (Qiagen) was performed to remove contaminating genomic DNA. The total RNA concentration was determined using a NanoDrop 2000 spectrometer (Thermo Scientific, Waltham, MA, USA), and then stored at −80°C until use, after dilution to 10 ng *μ*L^−1^.

### Analysis of PR, NaR, and ClR expression by quantitative real‐time PCR (qRT‐PCR)

The level of PR, NaR, and ClR gene expression was measured using qRT‐PCR. Synthesis of cDNAs was performed using the first‐strand cDNA synthesis kit (Enzynomics, Daejeon, Korea) according to manufacturer's instructions. Real‐time PCRs were carried out on a Step One Real‐Time PCR System (Applied Biosystems, Foster, CA, USA) with the SYBR Green Master Mix (Toyobo, Japan). The specific primers were designed by the Primer Express program (Applied Biosystems) and the primers used in this study are shown in Table S1. The PCR conditions were an initial denaturation step at 95°C for 5 min, followed by 40 cycles of amplification at 95°C for 30 sec, 60°C for 30 sec, and 72°C for 1 min. Finally, an additional step to establish the melting curve, in which the temperature was decreased from 95 to 65°C (0.3°C sec^−1^), was performed and quantified in triplicate. Relative quantification of gene expression values was calculated by the comparative critical threshold ∆∆C_T_ method (Livak and Schmittgen 2001). The PR, NaR, and ClR gene expressions were normalized to a 16S rRNA gene as an endogenous reference in the corresponding samples.

## Results and Discussion

### Diversity of PR‐containing flavobacteria

We screened the PR gene‐containing flavobacterial isolates from 322 bacterial strains of *Bacteroidetes*, which were isolated from diverse marine environments and deposited at the Marine and Extreme Bio‐resource Collection (www.megrc.re.kr/mebic/). In total, 16 microbial opsin sequences were obtained by PCR amplification using a primer set (PR‐F and ‐R) (Yoshizawa et al. 2012). The analysis of 16S rRNA sequences confirms the taxonomic position of all 16 strains as belonging to the family *Flavobacteriaceae* within the phylum *Bacteroidetes* (Table 1). Interestingly, PR homologue genes in 10 (62.5%) of 16 isolates were found from tidal flat sediment. It is not unreasonable that most microorganisms containing PR can utilize light energy and that enough light reaches the surface of the tidal flat (de la Torre et al. 2003). However, an understanding of the ecological role of PR homologue‐containing flavobacteria in the tidal flat sediment is lacking because most studies of the PR gene focused on bacterial strains isolated from marine surface water.

Since PR genes of marine isolates such as *Dokdonia* sp. MED134 and *Polaribacter* sp MED152 were found by whole genome sequencing (Gómez‐Consarnau et al. 2007), a study to find PR homologue‐containing flavobacteria was approached by PCR amplification using primers (Yoshizawa et al. 2012). In this study, all PR homologue‐containing flavobacteria were isolated from coastal, pelagic, and brackish waters, and they were affiliated with seven genera, *Winogradskyella*,* Polaribacter*,* Tenacibaculum*,* Gilvibacter*,* Kordia*,* Bizionia*, and *Flavobacterium*, among the 114 genera within the family of *Flavobacteriaceae*. In this study, additional PR homologue gene‐containing isolates were found, however, they were classified as members of the genera, *Aquimarina*,* Hoppeia*,* Sediminicola, Maribacter*, and *Flagellimonas* including previously reported genera such as *Nonlabens*,* Flavobacterium*, and *Dokdonia* (Fig. S1). The diversity of PR‐containing bacteria in this study is different from that of a previously reported study, although the same primers were used. This might have resulted from the difference of samples and it indicates that the diversity of PR homologue‐containing bacteria can be expanded by sampling the various habitats in marine environments.

The previous phylogenetic analyses of PR have mostly been performed on the basis of clone sequences (Béjà et al. 2000; de la Torre et al. 2003; Venter et al. 2004), and hence, there have been very few studies comparing the relationship between PR and 16S rRNA phylogenies from PR‐containing microorganisms. In this study, the phylogenetic analysis was compared between the 16S rRNA, and PR including proteins such as *Blh* (15,15′‐*β*‐carotene dioxygenase) and *CrtI* (phytoene dehydrogenase) involving retinal production (Figs. S2 and S3) (Misawa et al. 1995; Sabehi et al. 2005). The phylogenetic analysis of 16S rRNA demonstrated a discordance to the phylogenies of *CrtI* and *Blh* amino acid sequences as well as to a phylogeny of PR amino acid sequences. Particularly, the two genera, *Dokdonia* and *Nonlabens*, showed a formation of separate clades in PR genes, which might have resulted from a different evolutionary process. These incongruities clearly indicate that horizontal gene transfer of the PR gene and protein genes responsible for retinal production could have occurred. A similar argument was raised on the basis that PR clusters did not represent the phylogenetic clades of each genus (Yoshizawa et al. 2012). Frigaard et al. (2006) found PR genes among metagenomic sequences that bear an archaeal 16S rRNA sequence in the same metagenome fragment. They concluded that the PR gene can be horizontally transferred between domains, that is, between bacteria and archaea.

### Finding of NaR and ClR from PR‐containing strains and their phylogenetic relationship

Recently, novel rhodopsins such as NaR and ClR were reported (Inoue et al. 2013; Kwon et al. 2013; Yoshizawa et al. 2014). In order to discover novel NaR and ClR diversity from our 16 PR‐containing isolates, degenerate primers were designed to the conserved regions in the NaR and ClR proteins. The NaR genes were obtained from three isolates, *Nonlabens* sp. YIK11 and *Nonlabens* sp. MIC269 as well as *N*.* marinus* S1‐08^T^, for which PR and NaR genes were obtained before the publication of the presence of three rhodopsins based on whole‐genome sequencing. The ClR gene was obtained only from the YIK11 strain and this is likely the second reported isolate encoding three different rhodopsins after the first report for *N. marinus* S1‐08^T^. The occurrence of NaR and ClR from the16 PR‐containing flavobacteria was 19 and 13%, respectively, and the co‐occurrence of these two light‐driven rhodopsins with PR is not uncommon in marine flavobacteria.

**Figure 1:**
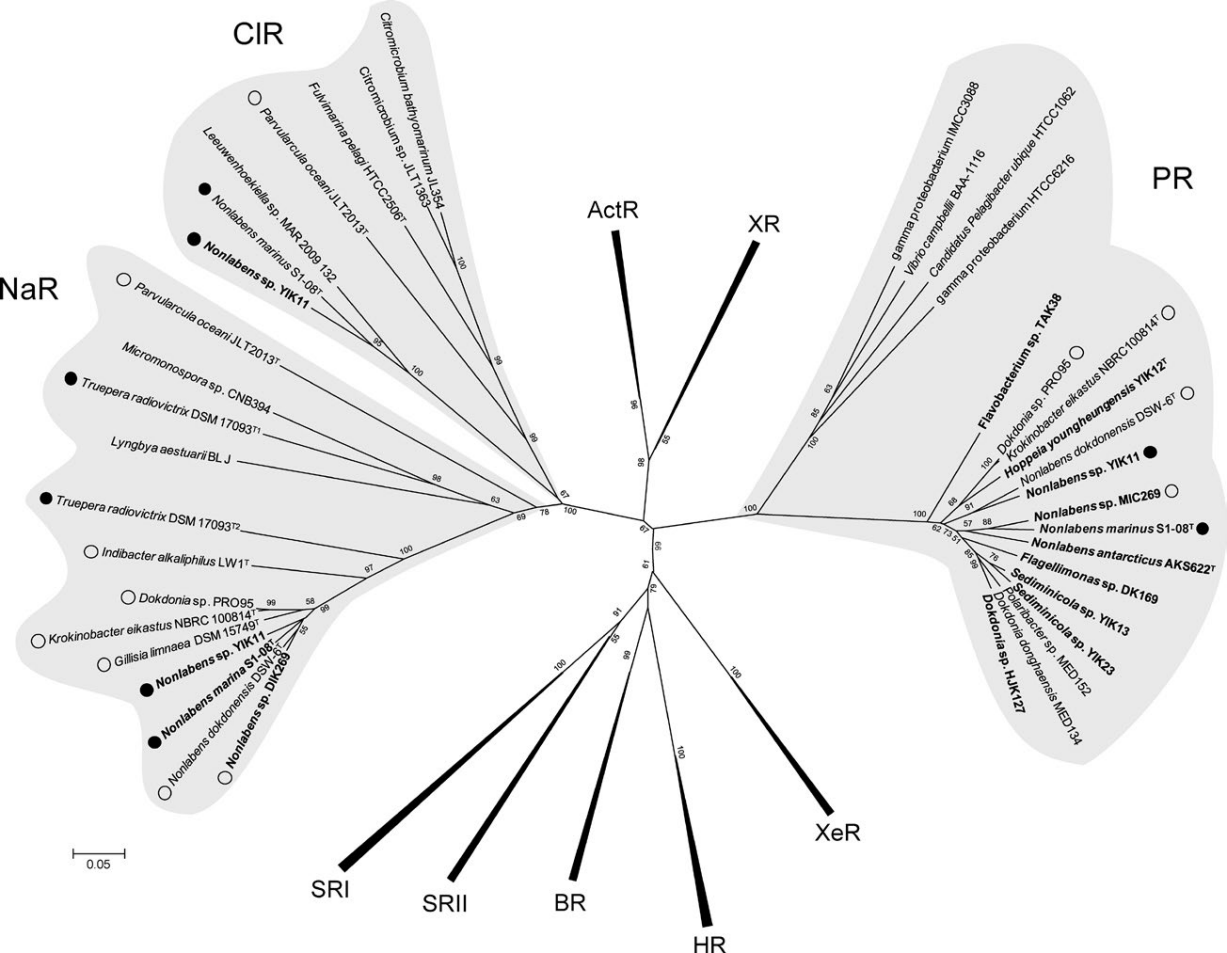
Phylogenetic tree of microbial rhodopsins. A tree inferred from 185 conserved amino acid positions was constructed using MEGA5. Bootstrap values for 1000 replicates are shown next to the branches. Boldface represents strains isolated in this study. Closed and open circles indicate strains containing three rhodopsins and two rhodopsins, respectively. ActR, Actinorhodopsin; BR, Bacteriorhodopsin; ClR, Chloride ion rhodopsin; HR, Halorhodopsin; NaR, Sodium ion rhodopsin; PR, Proteorhodopsin; SRI and II, Sensory rhodopsin I and II; XR, Xanthorhodopsin; XeR, Xenorhodopsin.

To further investigate the evolutionary relationship among PR, NaR, and ClR, and other rhodopsin family members, the conserved region from helix A to F was used for phylogenetic analysis. Phylogenetic analysis of microbial rhodopsins in our isolates revealed that one rhodopsin belongs to the PR‐clade (DTE motif) and the other rhodopsins were affiliated with NaR (NDQ motif) and ClR‐clades (NTQ motif) (Fig. 1). This result indicates that DTE, NDQ, or NTQ motif‐containing rhodopsins form a distinct phylogenetic group and they might function as homologs for proton, sodium, or chloride ion pumps, respectively. Recently, it was revealed that ClR evolved independently from halorhodopsin (HR), although ClR has a similar function as HR with regard to the light‐driven inward Clˉ pump (Yoshizawa et al. 2014). This finding is also supported by the YIK11 strain in this study. Interestingly, NTQ motif‐containing isolates were found only in seawater environments, including *Nonlabens marinus* S1‐08^T^ from seawater in the Pacific Ocean (Park et al. 2012); *Citromicrobium bathyomarinum* JL354 and *Citromicrobium* sp. JLT1363 from surface water in the South China Sea (Jiao et al. 2010; Zheng et al. 2011); *Fulvimarina pelagi* HTCC2506^T^ from the western Sargasso Sea (Kang et al. 2010); *Parvularcula oceanus* JLT2013^T^ from deep‐sea water of the southeastern Pacific Ocean (Li et al. 2014); *Leeuwenhoekiella* sp. MAR 2009 132 from phytoplankton (unpublished data), whereas YIK11 was first isolated from a sediment environment.

### Comparative analysis of full‐length rhodopsin gene sequences

The sizes of the PCR product for PR, NaR, and ClR genes partially obtained in this study were approximately 460, 657, and 453 bp, respectively. In an attempt to obtain the unknown 5′‐ and 3′‐end sequences of the three genes, DNA walking PCR was performed using gene‐specific primers. The full sequences of the PR, NaR, and ClR genes were used for the following analysis. PR homologue genes from YIK11, YIK13, HJK127, and S1‐08^T^ encoded peptides of 254, 242, 247, and 247 amino acid residues, respectively, sharing a sequence similarity of 70–72.4% (Table S2). NaR genes from YIK11, MIC269, and S1‐08^T^ encoded peptides of 279, 280, and 279 amino acid residues, respectively, sharing a sequence similarity range of 84.6–87.5% (Table S2). The ClR gene from YIK11 encoded a peptide of 272 amino acid residues. Also, the PR genes showed approximately 21 and 19% sequence identity with NaR and ClR, respectively (Table S2). A structure prediction program (DNASTAR) was applied to the amino acid sequences of PR, NaR, and ClR from representative flavobacterial microbial rhodopsins. Sequence alignment showed that PR, NaR, and ClR‐containing isolates have the same functional helix organization as the known rhodopsin proteins with highly conserved sequences between the helix C and G (Fig. S4). The several important amino acid residues necessary for energy generation were well conserved. For example, Lys 216, which binds retinal to helix G through a protonated Schiff base in bacteriorhodopsin was found to be conserved in YIK11 (Lys 233), YIK13 (Lys 224), HJK127 (Lys 223), and S1‐08^T^ (Lys 223) (Fig. S4). The Asp 85 and 96 residues, which act as a proton acceptor and donor, in the retinylidene Schiff base transfer during the PR photocycle (Braiman et al. 1988; Dioumaev et al. 2003), was found to be conserved in YIK11 (Asp 87 and Glu 98), YIK13 (Asp 88 and Glu 99), HJK127 (Asp 87 and Glu 98), and S1‐08^T^ (Asp 87 and Glu 98) (Table 2 and Fig. S4). The major member of the proton‐release group, the Arg 82 residue, was found to be conserved in YIK11 (Arg 84), YIK13 (Arg 85), HJK127 (Arg 84), and S1‐08^T^ (Arg 84) (Table 2 and Fig. S4). The NaR (KR2) residue appears to have the important features necessary for sodium ion pumping activity seen in H30, R109, N112, D116, and D251 (Inoue et al. 2013), and was found to be conserved at the same positions in MIC269, YIK11, and S1‐08^T^, and further, was found to be conserved in H29, R108, N111, D115, and D250 (Fig. S4). Taken together, the amino acid residues located in 85, 89, and 96 (BR numbering) play critical roles for conventional ion pumping activity, and PR, NaR, and ClR possess the DTE, NDQ, and NTQ motifs at the same positions, respectively (Table 2). Furthermore, a single amino acid in position 105 (in PR numbering) determines the wavelength of the absorbance maximum of PRs (Man et al. 2003). All detected microbial rhodopsins in this study carry a leucine or methionine residue at identical positions, which renders them as putative green‐light‐absorbing pigments. Therefore, the degree of sequence similarity and conserved functional features confirm that the newly isolated sequences are members of the microbial rhodopsin family.

**Table 2:** Amino acid sequence alignment of the C‐helix in various microbial rhodopsins

Rhodopsin type	Amino acid residue number in BR, PR, and NaR															
	BR	82	83	84	85	86	87	88	89	90	91	92	93	94	95	96
	PR	94	95	96	97	98	99	100	101	102	103	104	105	106	107	108
	NaR	109	110	111	112	113	114	115	116	117	118	119	120	121	122	123
BR		R	Y	A	**D**	W	L	F	**T**	T	P	L	L	L	L	**D**
PR		R	Y	I	**D**	W	L	L	**T**	V	P	L	L	I	C	**E**
NaR^a^		R	Y	L	**N**	W	L	I	**D**	V	P	M	L	L	F	**Q**
ClR^b^		R	Y	V	**N**	W	M	A	**T**	I	P	C	L	L	L	**Q**
XR		R	Y	V	**D**	W	L	L	**T**	V	P	L	L	T	V	**E**
ActR		R	Y	V	**D**	W	L	L	**T**	V	P	L	L	T	V	**E**
HR		R	Y	L	**T**	W	A	F	**S**	T	P	F	I	L	L	**A**
SRI		R	Y	V	**D**	W	V	V	**T**	T	P	L	L	V	G	**F**
SRII		R	Y	I	**D**	W	L	V	**T**	T	P	L	I	V	L	**Y**
PR^c^		R	Y	V	**D**	W	V or I	L	**T**	V	P	L	M	C	V	**E**
NaR^c^		R	Y	L	**N**	W	L	I or T	**D**	V	P	M	L	L	F	**Q**
ClR^c^		R	Y	V	**N**	W	M	A	**T**	I	P	C	L	L	V	**Q**

The bold indicates active site residues of microbial rhodopsins. This analysis was conducted as described by Inoue et al. (2013). PR, proteorhodopsin; BR, Bacteriorhodopsin; NaR, Sodium ion rhodopsin; ClR, chloride ion rhodopsin; XR, Xanthorhodopsin; ActR, Actinorhodopsin; SRI, sensory rhodopsin I; SRII, sensory rhodopsin II.

a *K. eikastus* NBRC 100814^T^ and *N. dokdonensis* DSW‐6^T^.

b *N. marinus* S1‐08^T^.

c Indicates the rhodopsins discovered during this study.

### Light‐driven pump activity of PR and putative NaR or ClR

**Figure 2:**
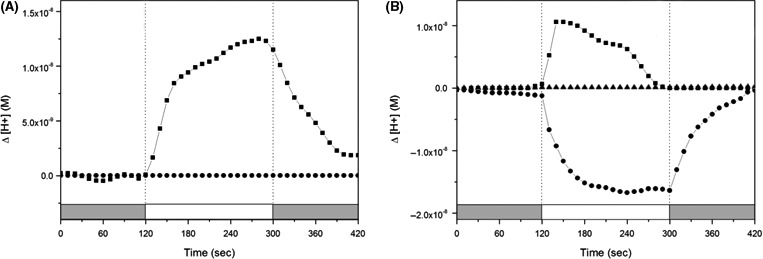
Light‐induced pump activity in native cell suspensions. (A) *Sediminicola* sp. YIK13, (B) *Nonlabens* sp. YIK11. Cells were collected at 48 h (data not shown) and 120 h growth. The changes in pH (initial pH: 7.0–7.2) were monitored for 2 min in the dark (gray regions), 3 min under light (>440 nm, white region), and 2 min in the dark, and this measurement was repeated three times. Squares, circles, and triangles indicate the change in protons outside of the cells without any addition, and with the addition of CCCP and CCCP + TPP ^+^, respectively. All the pump activities were measured under the same conditions.

We measured the light‐driven pump activity in native cell suspensions of three rhodopsin‐containing strains (highlighted with asterisk in Fig. S1). Pumping activity of each strain, (approximately 10^9^ cells mL^−1^) after 48 (early stationary growth phase) and 120 h (late stationary growth phase) incubation, was verified by measuring the pH difference with or without illumination. When cells of all strains at early growth phase were illuminated, they showed subtle or similar pumping activity with the late growth phase (data not shown). YIK13 containing only a PR gene showed a typical light‐induced proton pumping activity which was abolished in the presence of the protonophore carbonyl cyanide m‐chlorophenylhydrazone (CCCP) (Fig. 2A). YIK11 showed simultaneous light‐induced pumping activity of proton and putative sodium or chloride ions without or with the addition of CCCP, respectively, but the activity was completely abolished in the presence of both CCCP and tetraphenylphosphonium bromide (TPP^+^) (Fig. 2B). We used two or three rhodopsin‐containing strains, DSW‐6^T^ (PR and NQ), NBRC100814^T^ (KR1 and KR2), and S1‐08^T^ (NM‐R1, 2 and 3) as positive controls (Inoue et al. 2013; Kwon et al. 2013; Yoshizawa et al. 2014). DSW‐6^T^ and NBRC100814^T^ showed only the typical light‐driven proton pumping property (Fig. S5A and B). In contrast, S1‐08^T^ showed the same activity as YIK11 (Fig. S5C). Recently, biochemical analysis of the PR, NaR, and ClR proteins demonstrated that they have the ability to bind retinal and function as a light‐induced H^+^, Na^+^, and Cl^ˉ^ pump when expressed heterologously in an *Escherichia coli* overexpression system (Inoue et al. 2013; Yoshizawa et al. 2014). We showed light‐induced pumping activity of rhodopsins in the native cell suspensions, consistent with the phylogenetic positions and conserved functional residues.

### Effect of light on growth of *Nonlabens* sp. YIK11 strain

To investigate the effect of light on the cell growth and the expression of the rhodopsin genes, we selected the YIK11 strain, which encodes three different types of rhodopsins showing strong pumping activities of proton ions. YIK11 cells were cultured either in the nutrient‐limited or ‐enriched medium over a range of growth permissive salinities (optimum 3%) under continuous illumination (160 *μ*mol photons m^−2^ s^−1^) or continuous dark conditions. The cells collected at the exponential, early‐ and late stationary growth phases were counted by qNano, and the comparative expression ratio of relevant rhodopsin genes were quantitatively measured by real‐time PCR (qRT‐PCR). YIK11 cells grown in nutrient‐enriched ZB media with different levels of salinity showed significantly higher yields in the dark as compared to the cells grown under illumination (Fig. S3A–C). Significant differences were observed in the exponential and stationary growth phases, and cell yields in the dark were about 1.5–2.8 times higher than that of cells under illumination, and this trend continued through the late stationary growth phase (Fig. S3A–C). To further investigate whether the growth rate was affected by the illumination level, YIK11 cells were grown in the nutrient‐enriched optimal ZB medium with different light intensities. The cell yield of YIK11 grown under 25 *μ*mol photons m^−2^ s^−1^ illumination was maximally about 2.3 times higher than that under 160 *μ*mol photons m^−2^ s^−1^ illumination. However, this value is still lower than that of dark culture (Fig. S3D). When culture was changed from light to dark condition after 46 h, growth was initiated and reached a maximal yield within 26 h (Fig. S3D). These data imply that high levels of irradiance cause photoinhibition on the growth of *Nonlabens* sp. YIK11, possessing three different types of rhodopsins. Earlier observations for *Halobacterium salinarum* and *Salinibacter ruber* showed that proton pumping by BR and XR causes inhibition of respiration (Boichenko et al. 2006). This might inhibit cell growth, since the consumption of organic substrates slows because of the inhibition of respiration. Recently, Na et al. (2015) reported that the inhibition of growth was caused by intracellular reactive oxygen species (ROS) as the ATP levels increased. The high intensity of light enhanced the ATP production in the cells of YIK11 owing to the light‐driven pumping rhodopsins and it possibly caused the inhibition of growth as the ROS increased. The results of the growth experiments in this study showed similar growth inhibition pattern due to stimulatory effect of all three rhodopsins, consistent with that reported for *Vibrio campbellii* BAA‐1116, studied using one PR (Wang et al. 2012). Similarly, *Psychroflexus torques* ATCC 700755^T^ has shown enhanced growth yield under a lower intensity than under high illumination, but no enhancement under dark or osmotic pressure conditions (Feng et al. 2013). We suppose that the continuous exposure to light stress may lead to the accumulation of ROS‐related damage to cellular components and inhibition of respiration, even if it contains enough nutrients.

In nutrient‐limited seawater culture containing 0.14 mmol L^−1^ carbon, YIK11 did not significantly change in cell yields under light and dark conditions, that is oppositely described in Gómez‐Consarnau et al. (2007) (Fig. S3E). Similar results were obtained in the culture containing 0.39 and 0.74 mmol L^−1^ carbon (data not shown). PR‐containing marine flavobacteria showed light‐stimulated growth at low‐carbon concentrations (Gómez‐Consarnau et al. 2007; Yoshizawa et al. 2014) and in a nutrient‐enriched medium supplemented with a high concentration of salts (Feng et al. 2013). However, other PR‐containing bacteria did not show any significant enhancement of growth rate or yield by light, which is consistent with our findings (Giovannoni et al. 2005; Stingl et al. 2007; González et al. 2008; Steindler et al. 2011; Riedel et al. 2013).

**Figure 3:**
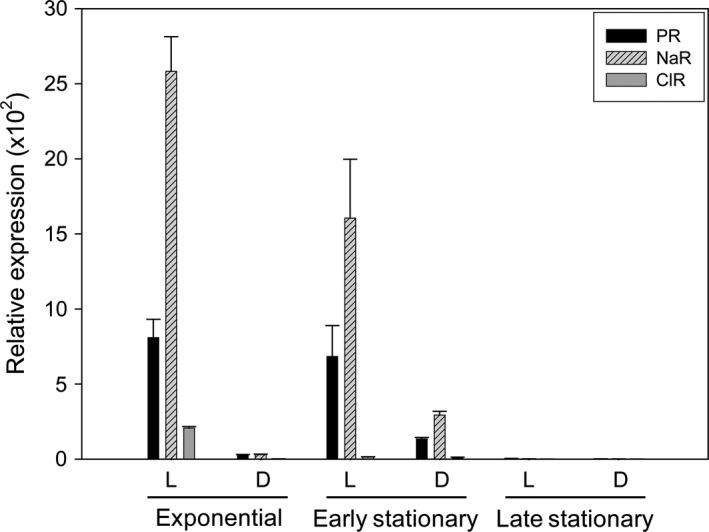
Relative expression levels of PR, NaR, and ClR genes in *Nonlabens* sp. YIK11 grown in nutrient‐limited medium (see Fig. S6E). The cultures were collected at exponential (24 h), early (36 h), and late (72 h) stationary phases for RNA extraction. The PR, NaR, and ClR expression levels were determined by qRT‐PCR analysis and calculated based on the ∆∆C_T_ method (Livak and Schmittgen 2001). The 16s rRNA gene was used as control. L, light at 160 *μ*mol photons m^−2^ s^−1^; D, darkness; PR, Proteorhodopsin. Error bars denote standard deviations for triplicate cultures; when not visible, error bars are within symbols.

The qRT‐PCR comparison between cells cultured under illumination and in the dark indicated that three rhodopsin genes are highly induced when they are incubated in the presence of light or in the absence of sufficient nutrients (Fig. 3). However, the expression levels of the NaR gene were much higher than those of the PR or ClR genes under the same culture conditions as mentioned above. The relative expression levels of three rhodopsin genes were highest during the exponential phase (NaR, 2582 ± 232; PR, 809 ± 122; ClR, 207 ± 10), then gradually declined from the early stationary phase, and finally, they became very low at the late stationary phase (Fig. 3). In contrast, when cells were grown with sufficient nutrients, different salt concentrations or illumination levels had no significant effect on the expression levels of the three rhodopsin genes (data not shown). This result suggests that the expression levels of the DTE, NDQ, and NTQ motif‐containing rhodopsin genes in *Nonlabens* sp. YIK11 correlate positively with illumination, but negatively with nutrient levels, which is consistent with the previous report on PR or NaR gene expression (Gómez‐Consarnau et al. 2007; Lami et al. 2009; Kimura et al. 2011; Wang et al. 2012; Kwon et al. 2013). On the basis of those results, we concluded that light has a positive impact on the relative expression levels of the three rhodopsin genes in the flavobacterium, *Nonlabens* sp. YIK11, but with no apparent positive impact on growth.

## Conclusions

In this study, we report the presence and function of one or more microbial rhodopsins in rhodopsin‐containing flavobacteria from diverse marine environments, mainly in the sediment. The RT‐PCR analysis demonstrated that three different rhodopsin genes are expressed by strain YIK11 and it implies that rhodopsin‐mediated phototrophy plays an important role in this organism. A light‐induced translocation activity showed that one or more rhodopsin proteins are functional within sediment. However, we were not able to detect any enhanced growth rate of YIK11 cells when cultured in the light rather than in the dark. Fuhrman et al. (2008) suggested that PR might be used as a survival mechanism under harsh conditions. Possibly, the physiological function of light‐induced rhodopsin activity for energy acquisition might be better understood through studies under starvation or similar stress conditions found in the natural environment.

## Conflict of Interest

None declared.

## Supporting information


**Figure S1.** Phylogenetic tree of PR amino acid sequences (155 positions).

**Figure S2.** Phylogenetic tree of Blh amino acid sequences (176 positions).

**Figure S3.** Phylogenetic tree of CrtI amino acid sequences (376 positions).

**Figure S4.** Multiple alignments of PR, NaR, and ClR amino acid sequences.

**Figure S5.** Light‐induced pump activity in native cell suspensions.

**Figure S6.** Growth of *Nonlabens* sp. YIK11 cultured in different nutrient concentrations and light conditions.

**Table S1.** List of primers used in this study for PCR and qPCR.

**Table S2.** Sequence identities among representative rhodopsins from diverse flavobacteria strains.
