# Supplementary figures and images for: Diversity and functional analysis of light‐driven pumping rhodopsins in marine Flavobacteria
Source: Microbiologyopen. 2015 Dec 13;5(2):212–23. doi: 10.1002/mbo3.321 (PMC4831467; doi:10.1002/mbo3.321)

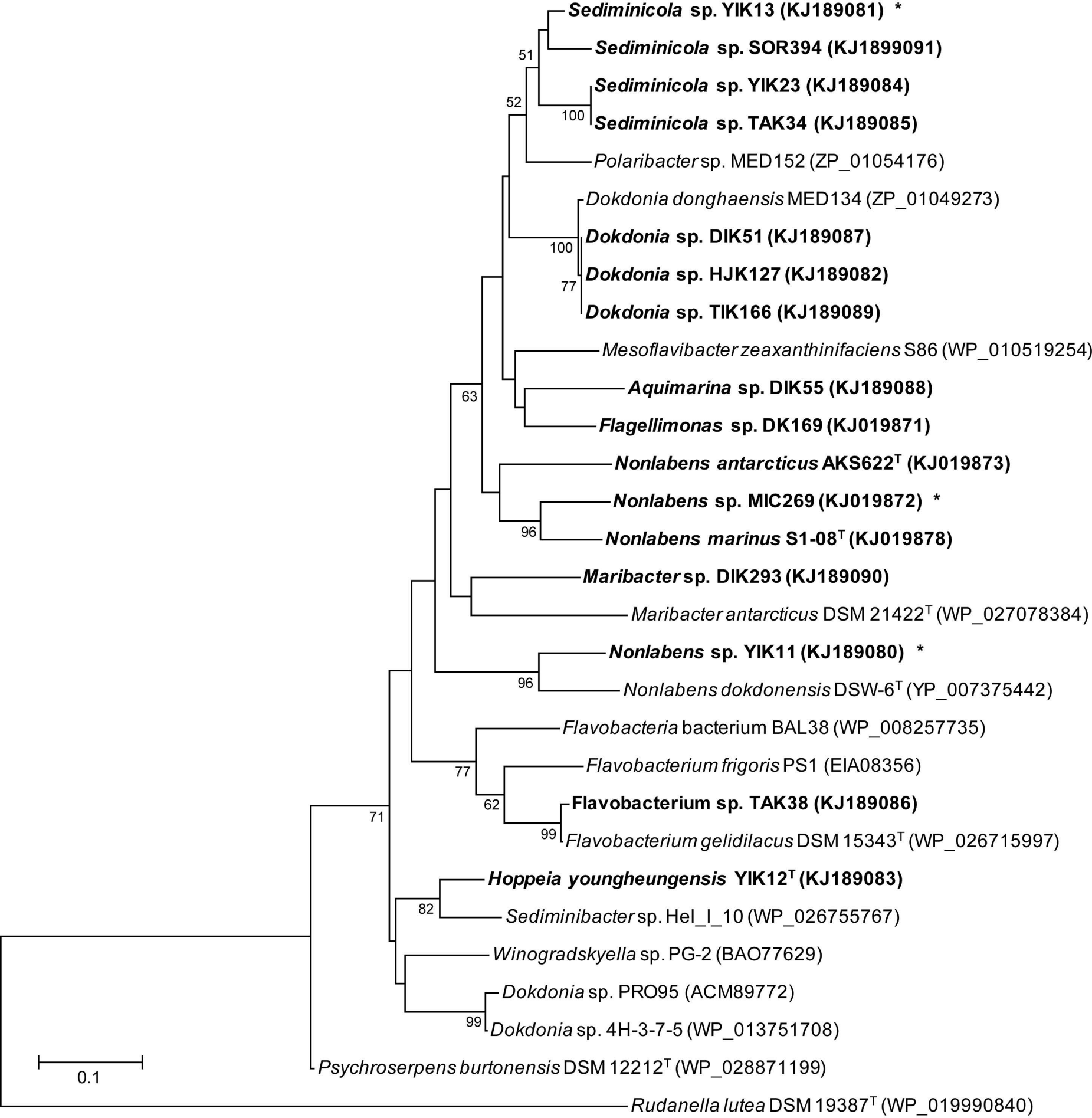

Supplement: Supplementary file 1 — Figure S1. Phylogenetic tree of PR amino acid sequences (155 positions). [file MBO3-5-212-s001.pdf]

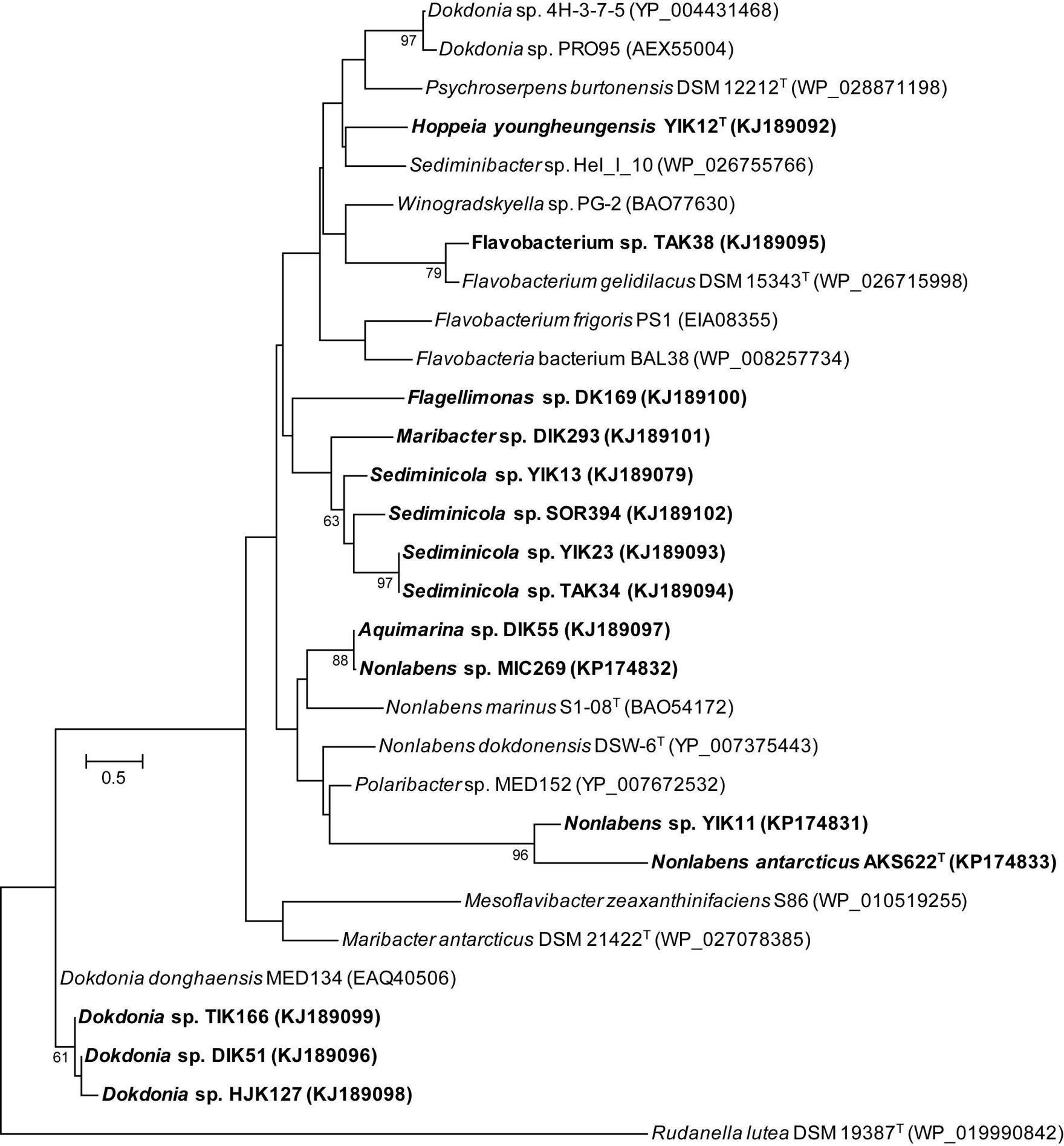

Supplement: Supplementary file 2 — Figure S2. Phylogenetic tree of Blh amino acid sequences (176 positions). [file MBO3-5-212-s002.pdf]

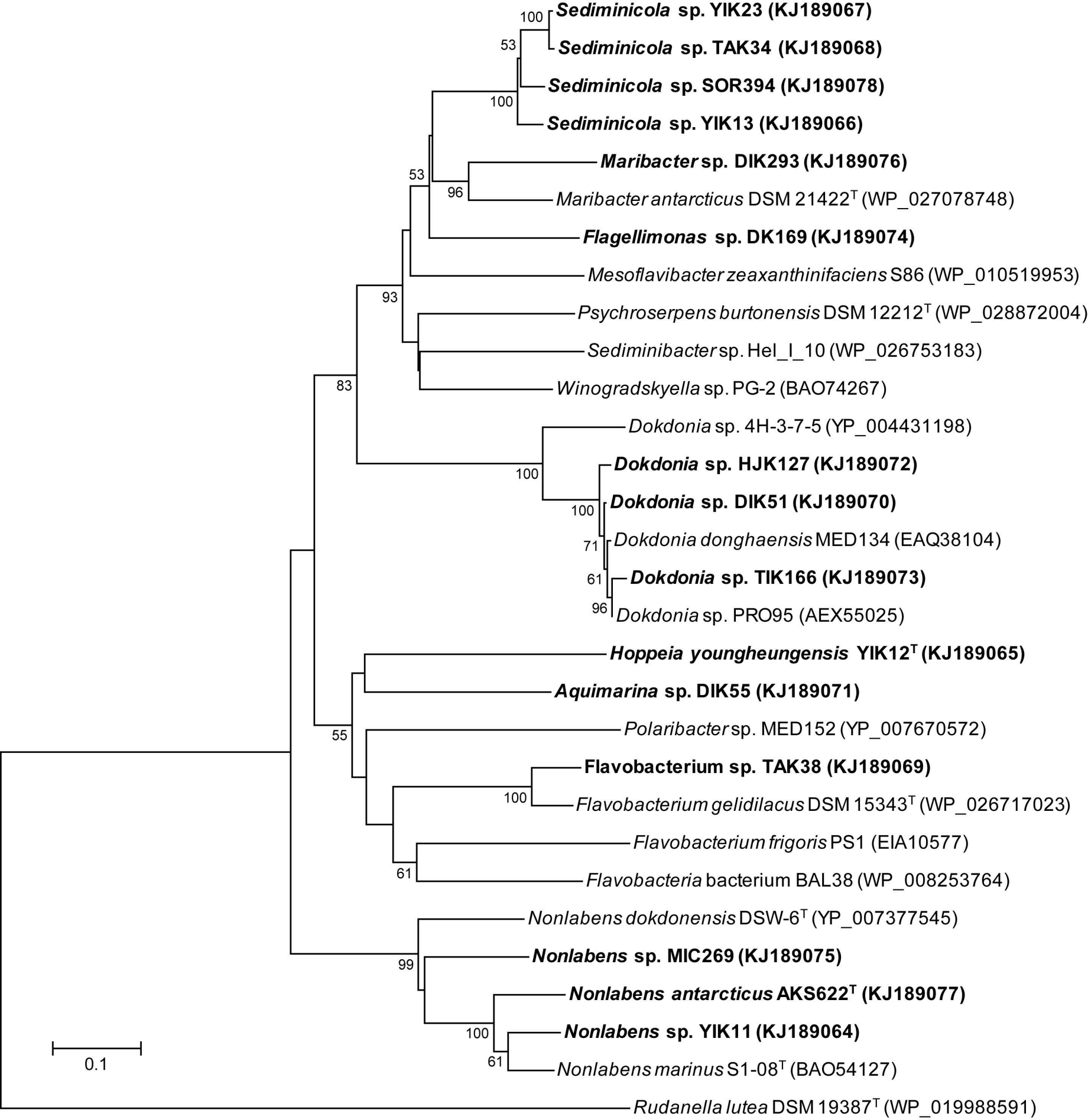

Supplement: Supplementary file 3 — Figure S3. Phylogenetic tree of CrtI amino acid sequences (376 positions). [file MBO3-5-212-s003.pdf]

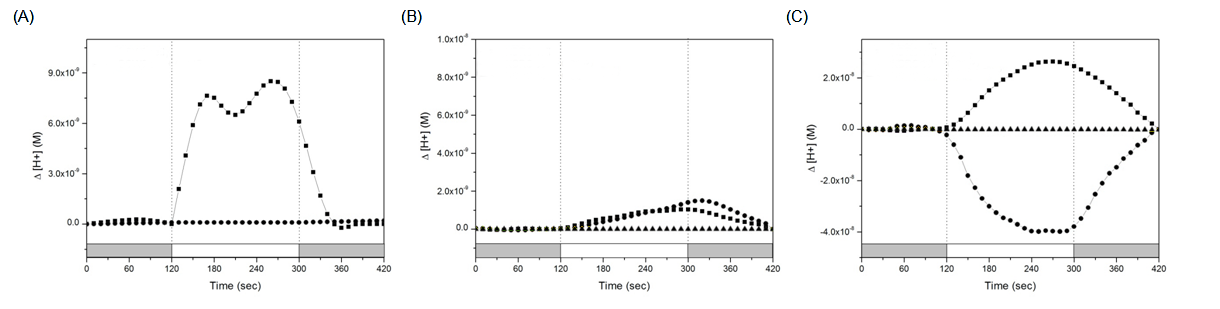

Supplement: Supplementary file 5 — Figure S5. Light‐induced pump activity in native cell suspensions. [file MBO3-5-212-s005.tif]

(A)

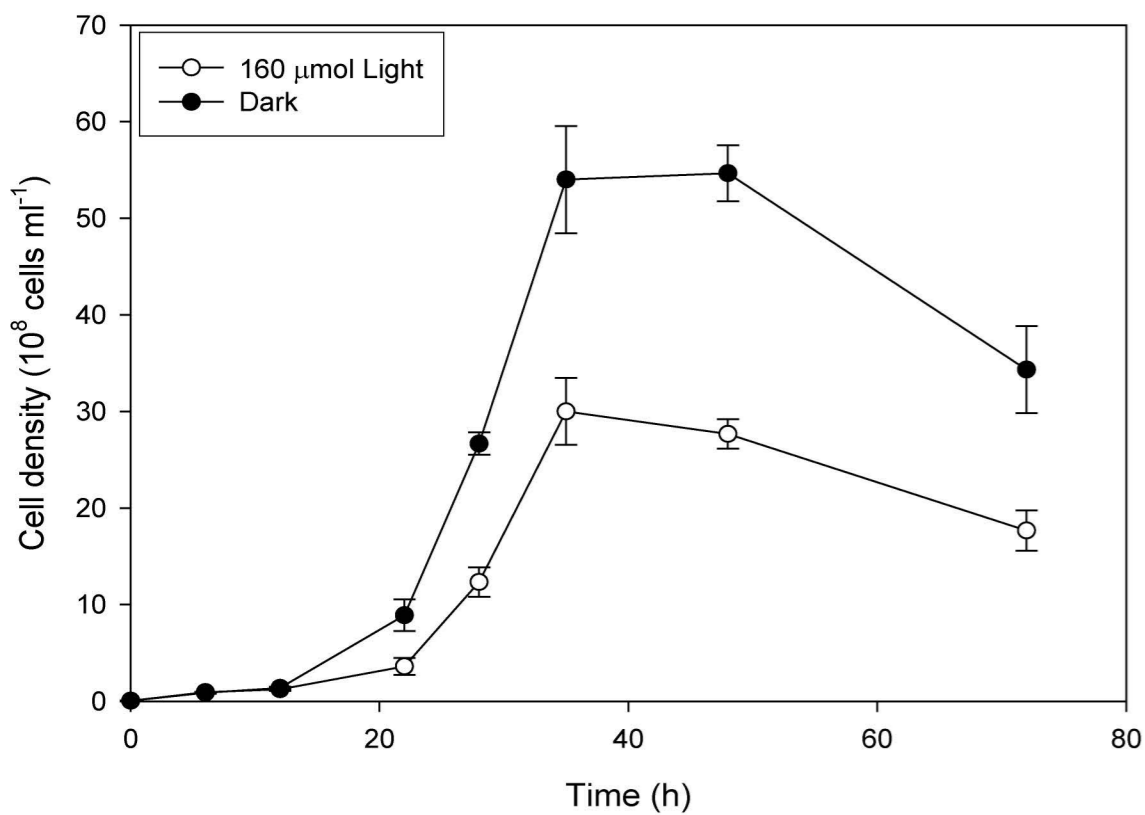

(B)

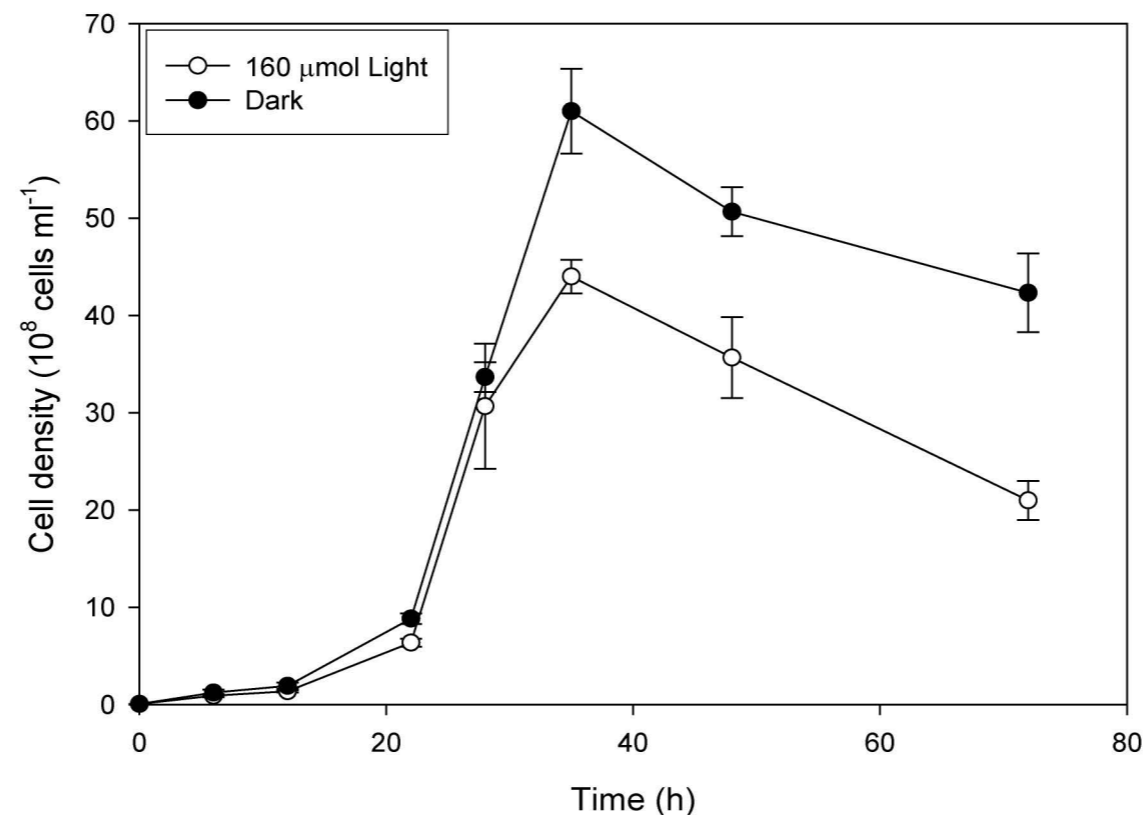

(C)

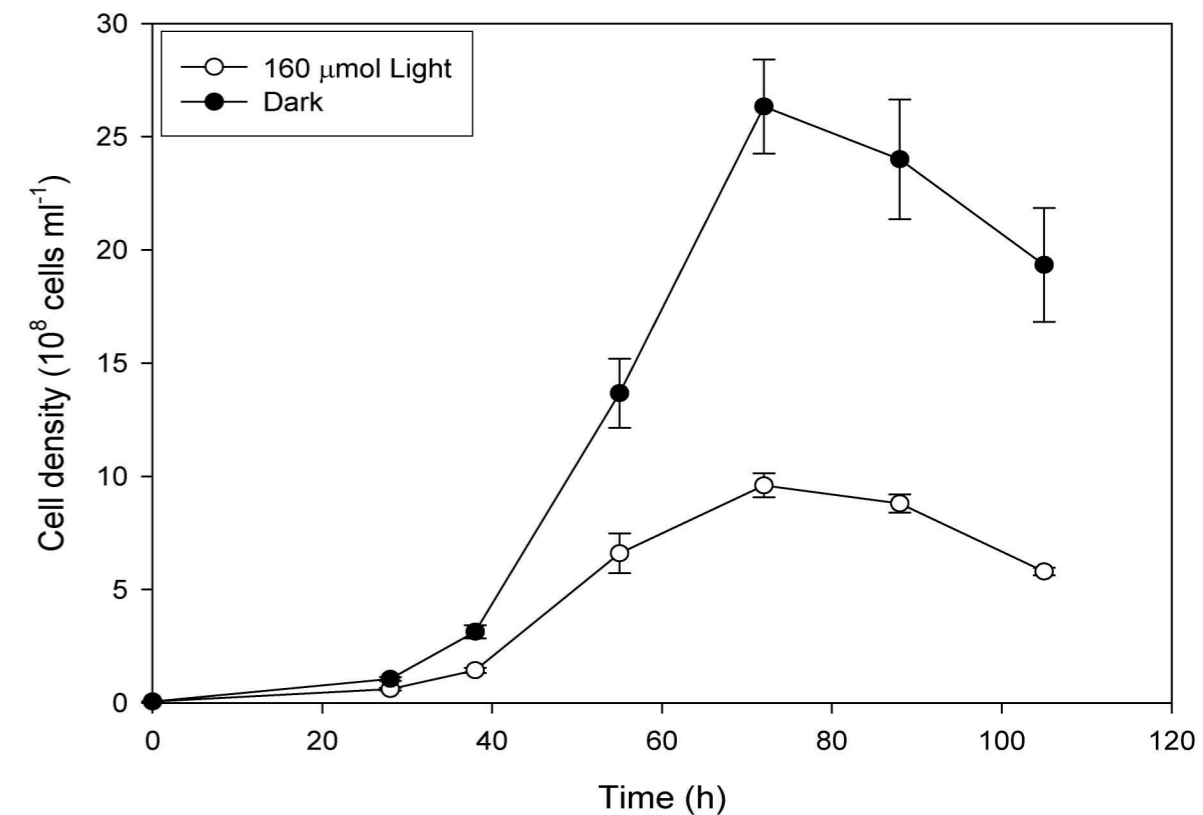

(D)

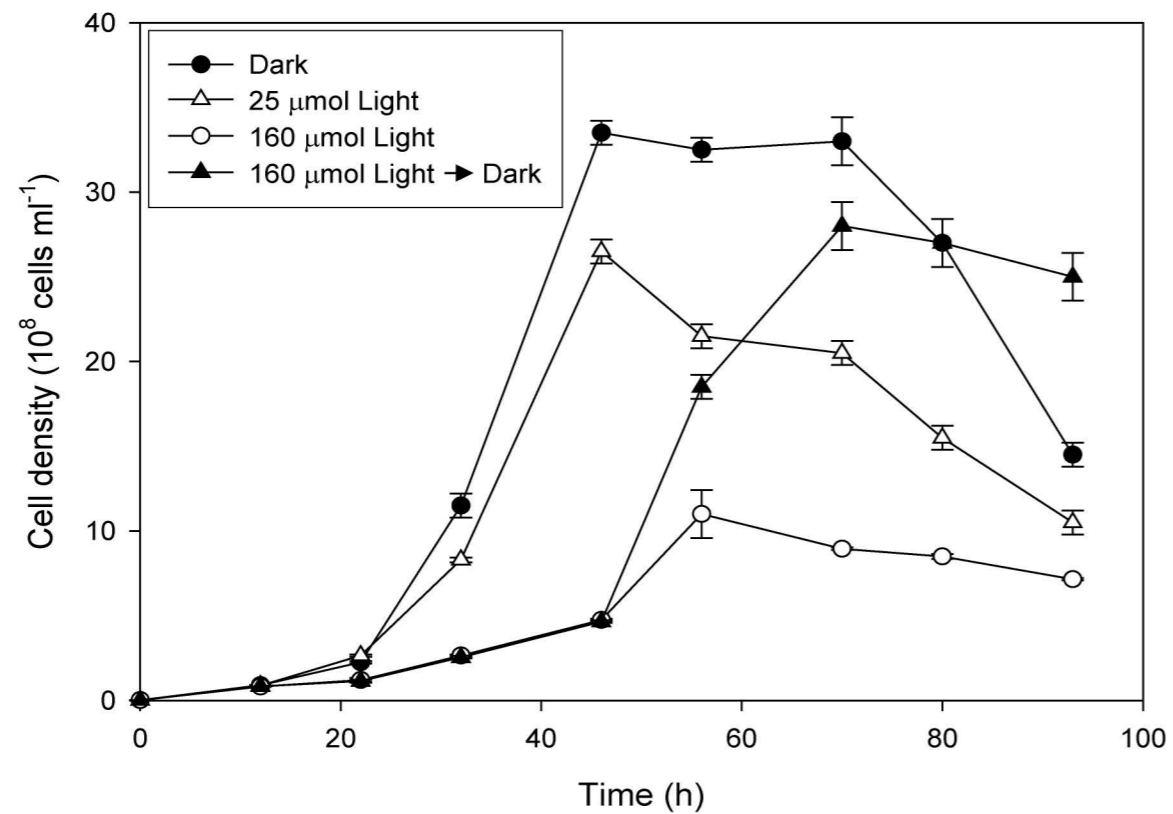

(E)

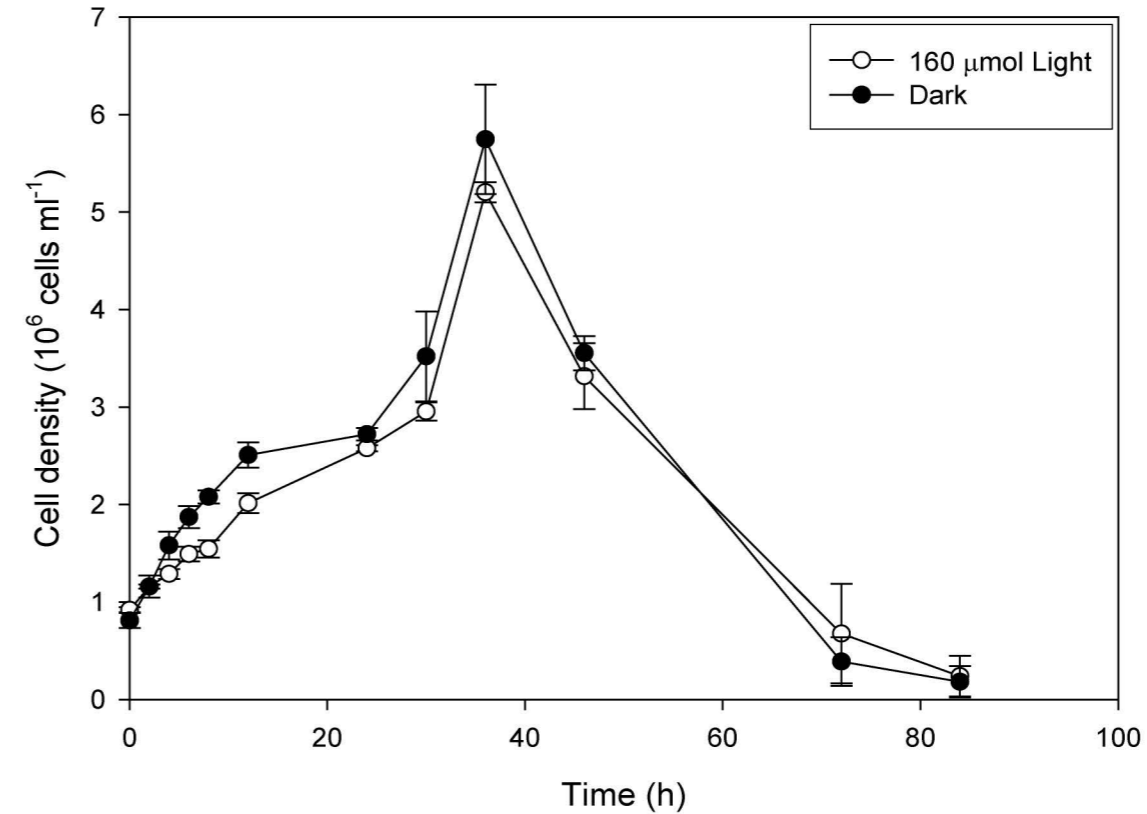

Supplement: Supplementary file 6 — Figure S6. Growth of Nonlabens sp. YIK11 cultured in different nutrient concentrations and light conditions. [file MBO3-5-212-s006.pdf]
