# Supplementary material for: Diversity and functional analysis of light‐driven pumping rhodopsins in marine Flavobacteria
Source: Microbiologyopen. 2015 Dec 13;5(2):212–23. doi: 10.1002/mbo3.321 (PMC4831467; doi:10.1002/mbo3.321)
Supplement: Supplementary file 4 — Figure S4. Multiple alignments of PR, NaR, and ClR amino acid sequences. [file MBO3-5-212-s004.pdf]

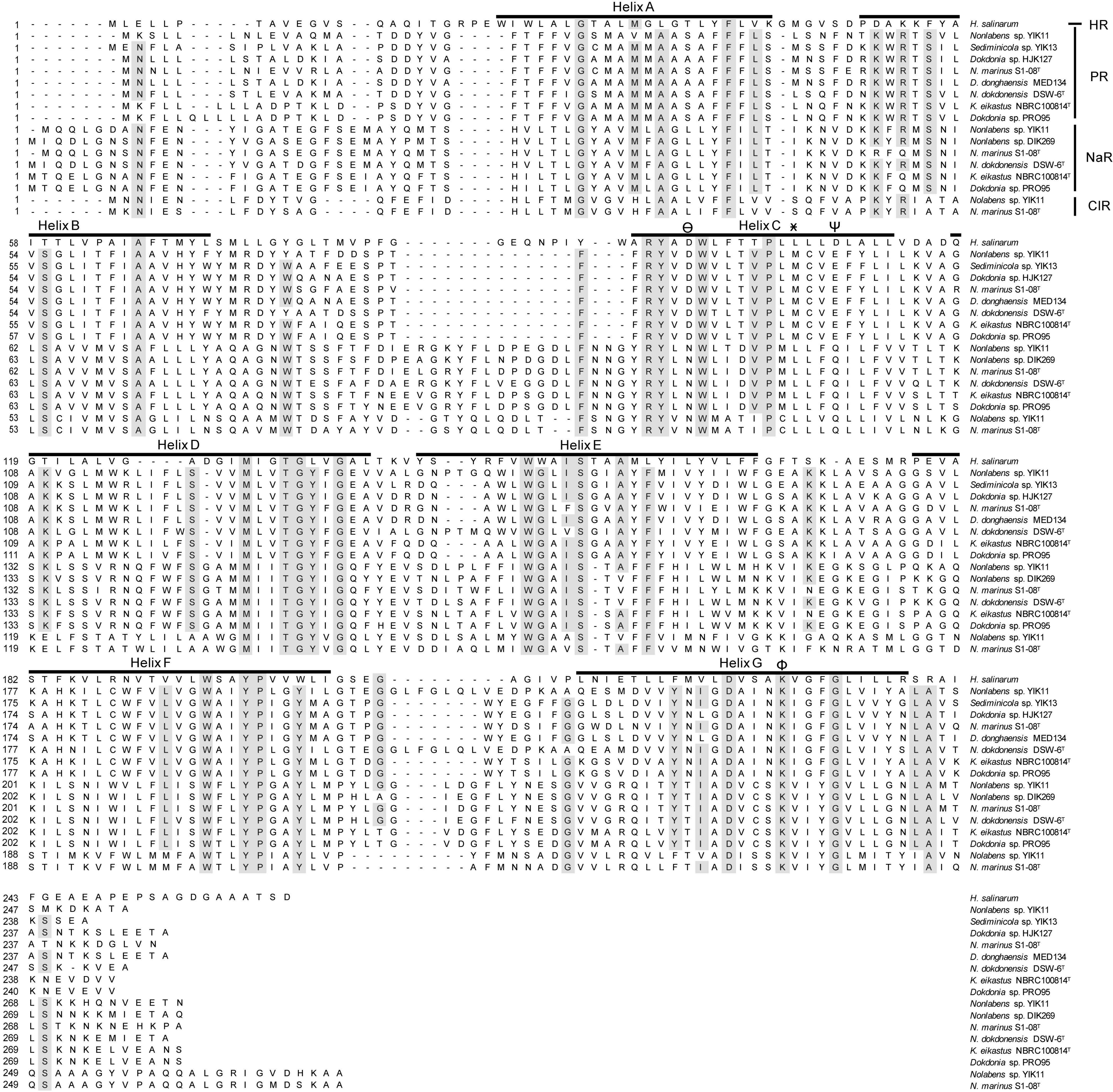

Helix A
Helix B
Helix C
Helix D
Helix E
Helix F
Helix G
Θ
Ψ
Φ
HR
PR
NaR
CIR
H. salinarum
Nonlabens sp. YIK11
Sediminicola sp. YIK13
Dokdonia sp. HJK127
N. marinus S1-08T
D. donghaensis MED134
N. dokdonensis DSW-6T
K. eikastus NBRC100814T
Dokdonia sp. PRO95
Nonlabens sp. DIK269
Nolabens sp. YIK11
