# Supplementary material for: Diversity and functional analysis of light‐driven pumping rhodopsins in marine Flavobacteria
Source: Microbiologyopen. 2015 Dec 13;5(2):212–23. doi: 10.1002/mbo3.321 (PMC4831467; doi:10.1002/mbo3.321)
Supplement: Supplementary file 7 — Table S1. List of primers used in this study for PCR and qPCR. [file MBO3-5-212-s007.doc]

| **Supplementary Table S1. List of primers used in this study for PCR and qPCR** | | | | | |
| --- | --- | --- | --- | --- | --- |
| A. Primers for PCR amplification of each genes | | | | | |
| Gene | Forward primer (5' → 3') |  | Reverse primer (5' → 3') | Product size (bp) | Reference |
| NaR-1 | ACNYTNGGNTAYGCNGTNATG |  | CANACRTCNGCDATNGTRTA | 660 | In this study |
| NaR-2 | ACNGGNTAYATHGGNCAR |  |  | 310 |  |
| ClR | GTNATGGYNWSNGCNGGNYTN |  | RTANGCDATNGGTRANAR | 460 |  |
| *Blh* | TTYGGNATHYTNCAYGGNGCNAAYG |  | GNGGRAANGTDATNGCNGCNARRAA | 740 |  |
| *CrtI* | TGGTAYTGGATGCCHGATRTNTTYGA |  | GMDGGNGGHACDCCNGGNCC | 1250 |  |
| PR | GAYTAYGTWGSWTTYACDTTYTTTGTRGG |  | GCCCAWCCHACWARWACRAACCARCATA | 460 | Yoshizawa *et al*., 2012 |
|  |  |  |  |  |  |
| B. Primers for real-time qPCR analysis of each gene transcript expression | | | | | |
| Gene | Forward primer (5' → 3') |  | Reverse primer (5' → 3') |  |  |
| 16S | TGCAAGCGTTATCCGGAAT |  | GCAAACTGTCCGAAGAAAAGC |  | In this study |
| PR | TTTGTAGGTAGTATGGCCGTGATG |  | CAGACGTTCTCCATTTTGTATTGAA |  |  |
| NaR | ACATAGGTGCTACAGAAGGCTTTTC |  | AGCATCACGGCATAGCCTAAA |  |  |
| ClR | CTGATGGTGTTGTATTGAGACAAGTCT |  | GCTTGTTGAGCAGGTACGTAACC |  |  |
