# Supplementary material for: Diversity and functional analysis of light‐driven pumping rhodopsins in marine Flavobacteria
Source: Microbiologyopen. 2015 Dec 13;5(2):212–23. doi: 10.1002/mbo3.321 (PMC4831467; doi:10.1002/mbo3.321)
Supplement: Supplementary file 8 — Table S2. Sequence identities among representative rhodopsins from diverse flavobacteria strains. [file MBO3-5-212-s008.doc]

| **Supplementary Table S2. Sequence identities amongst representative rhodopsins from diverse flavobateria strains. Percent identities were calculated by a** | | | | | | | | | | | | | | | | | | | |
| --- | --- | --- | --- | --- | --- | --- | --- | --- | --- | --- | --- | --- | --- | --- | --- | --- | --- | --- | --- |
| **computer program (MEGALIGN).** The numbering is based on Fig. S4 (from top to bottom). | | | | | | | | | | | | | | | | | | | |
| 1 | 2 | 3 | 4 | 5 | 6 | 7 | 8 | 9 | 10 | 11 | 12 | 13 | 14 | 15 | 16 | 17 |  | Strains | Remarked by |
|  | 18.5 | 15.7 | 18.2 | 17.9 | 18.2 | 18.2 | 16.4 | 16.3 | 19.5 | 18.7 | 19.1 | 18.7 | 20.6 | 20.6 | 12.6 | 14.9 | 1 | *H. salinarum* | Oesterhelt and Stoeckenius, 1971 |
|  |  | 72.3 | 70 | 72.4 | 70 | 86.6 | 70.9 | 69.5 | 21.3 | 21.7 | 18.1 | 19.3 | 22.4 | 21.3 | 19.3 | 20.1 | 2 | *Nonlabens* sp. YIK11 | In this study |
|  |  |  | 84.3 | 79.8 | 84.3 | 73.6 | 74.8 | 73.1 | 21.1 | 20.7 | 20.7 | 23.1 | 20.7 | 19.8 | 21.5 | 19.8 | 3 | *Sediminicola* sp. YIK13 |
|  |  |  |  | 79.7 | 99.6 | 68.4 | 73.4 | 71.5 | 19.8 | 23.1 | 19.8 | 22.7 | 21.1 | 21.1 | 20.2 | 19.8 | 4 | *Dokdonia* sp. HJK127 |
|  |  |  |  |  | 79.7 | 69.9 | 72.5 | 71.1 | 17.9 | 20.3 | 18.3 | 20.3 | 21.1 | 21.1 | 19.5 | 19.1 | 5 | *N. marinus* S1-08^T^ |
|  |  |  |  |  |  | 68.4 | 73.4 | 71.5 | 19.8 | 23.1 | 19.8 | 22.7 | 21.1 | 21.1 | 20.2 | 19 | 6 | *D. donghaensis* MED134 | Gómez-Consarnau *et al*., 2007 |
|  |  |  |  |  |  |  | 68.9 | 67.5 | 21.7 | 19.8 | 20.6 | 20.6 | 22.5 | 22.5 | 19 | 18.6 | 7 | *N. dokdonensis* DSW-6^T^ | Kwon *et al*., 2013 |
|  |  |  |  |  |  |  |  | 96.7 | 21.3 | 21.3 | 20.1 | 20.5 | 20.5 | 20.5 | 17.6 | 17.6 | 8 | *K. eikastus* NBRC100814^T^ | Inoue *et al*., 2013 |
|  |  |  |  |  |  |  |  |  | 21.5 | 21.5 | 20.3 | 20.7 | 20.3 | 20.3 | 17.1 | 17.5 | 9 | *Dokdonia* sp. PRO95 | Riedel *et al*., 2010 |
|  |  |  |  |  |  |  |  |  |  | 84.6 | 87.5 | 84.6 | 82.1 | 81.4 | 35.3 | 34.9 | 10 | *Nonlabens* sp. YIK11 | In this study |
|  |  |  |  |  |  |  |  |  |  |  | 84.2 | 92.8 | 79.6 | 78.9 | 34.2 | 34.2 | 11 | *Nonlabens* sp. DIK269 |
|  |  |  |  |  |  |  |  |  |  |  |  | 82.1 | 82.4 | 81 | 33.8 | 32.7 | 12 | *N. marinus* S1-08^T^ |
|  |  |  |  |  |  |  |  |  |  |  |  |  | 78.9 | 78.1 | 34.9 | 35.7 | 13 | *N. dokdonensis* DSW-6^T^ | Kwon *et al*., 2013 |
|  |  |  |  |  |  |  |  |  |  |  |  |  |  | 98.2 | 34.9 | 33.5 | 14 | *K. eikastus* NBRC100814^T^ | Inoue *et al*., 2013 |
|  |  |  |  |  |  |  |  |  |  |  |  |  |  |  | 35.3 | 33.8 | 15 | *Dokdonia* sp. PRO95 |
|  |  |  |  |  |  |  |  |  |  |  |  |  |  |  |  | 86 | 16 | *Nonlabens* sp. YIK11 | In this study |
|  |  |  |  |  |  |  |  |  |  |  |  |  |  |  |  |  | 17 | *N. marinus* S1-08^T^ | Yoshizawa *et al*., 2014 |
